# A Contactless Low-Carbon Steel Magnetostrictive Torquemeter: Numerical Analysis and Experimental Validation

**DOI:** 10.3390/s24216949

**Published:** 2024-10-29

**Authors:** Carmine Stefano Clemente, Claudia Simonelli, Nicolò Gori, Antonino Musolino, Rocco Rizzo, Marco Raugi, Alessandra Torri, Luca Sani

**Affiliations:** 1 Department of Energy, Systems, Territory and Construction Engineering, University of Pisa, 56122 Pisa, Italy; nicolo.gori@phd.unipi.it (N.G.); antonino.musolino@unipi.it (A.M.); rocco.rizzo@unipi.it (R.R.); marco.raugi@unipi.it (M.R.); luca.sani@unipi.it (L.S.); 2 Avio Aero, A GE Aerospace Company, 10040 Rivalta di Torino, Italy; alessandra.torri@avioaero.it

**Keywords:** contactless torquemeter, magnetostrictive sensor, Villari effect

## Abstract

Torque measurement is a key task in several mechanical and structural engineering applications. Most commercial torquemeters require the shaft to be interrupted to place the sensors between the two portions of the shaft where a torque has to be measured. Contactless torquemeters based on the inverse magnetostrictive effect represent an effective alternative to conventional ones. Most known ferromagnetic materials have an inverse magnetostrictive behavior: applied stresses induce variations in their magnetic properties. This paper investigates the possibility of measuring torsional loads applied to a shaft made of ferromagnetic steel S235 through an inverse magnetostrictive torquemeter. It consists of an excitation coil that produces a time-varying electromagnetic field inside the shaft and an array of sensing coils suitably arranged around it, in which voltages are induced. First, the system is analyzed both in unloaded and loaded conditions by a Finite Element Method, investigating the influence of relative positions between the sensor and the shaft. Then, the numerical results are compared with the experimental measurements, confirming a linear characteristic of the sensor (sensitivity about 0.013 mV/Nm for the adopted experimental setup) and revealing the consistency of the model used. Since the system exploits the physical behavior of a large class of structural steel and does not require the introduction of special materials, this torquemeter may represent a reliable, economical, and easy-to-install device.

## 1. Introduction

Many industrial applications (e.g., those in the Industry 4.0 environment) require low-cost, highly reliable, safe, and environmentally compatible torque sensors. For example, it is often necessary to measure the torque in a drive shaft of marine engines or for wheel or train transport. Furthermore, the measurement of the tremendous torque transmitted from a gas turbine to the propeller blades in the aeronautical field is now mandatory. In this framework, a contactless torquemeter which does not alter the sensed system by introducing discontinuity on the shaft, as conventional torquemeters do, is very desirable. Furthermore, it can be used for real-time static and dynamic measurement tasks and allows the monitoring of the system to prevent non-compliant operating conditions [1].

The most common types of industrial torque sensors are strain gauge based. These use strain gauges, ±45° bonded to the shaft, to detect every small deformation caused by torque. As the shaft twists, the gauges measure the strain, converting it into an electrical signal that correlates to the applied torque. These sensors are quite widely used for their precision and reliability but can be sensitive to environmental conditions, and their performance may degrade over time. Usually, the strain gauges, when used with rotating shafts, are wirelessly coupled with the stationary frame [2].

To overcome the drawbacks related to conventional torquemeters, in the last decades, a lot of effort has been spent in applications such as contactless torque sensors. One the best candidates to act in this field is the magnetostrictive sensor, which is based on the intrinsic property of magnetostriction in some ferromagnetic materials. In particular, in such kinds of devices, the inverse magnetostrictive effect, known as the Villari effect, is employed. It exploits the change in the magnetic permeability of the material which undergoes mechanical external stresses. A sample of the material has to be magnetically excited by a known source to determine the magnetic characteristic variations; this is a common feature of other smart materials (e.g., magnetorheological fluids [3,4]). There are two ways to obtain this scope. If measurements are made on materials with a sufficient residual magnetization, passive devices can be conceived. The property of these materials of keeping residual magnetization is exploited, as if they were permanent magnets. This feature requires magnetically encoding the material to be sensed, which is expensive; moreover, not all materials retain their magnetization over long periods. In addition, such an operation makes it more difficult to retrofit existing systems with a magnetostrictive sensor because the material to be sensed, e.g., a shaft of the gas turbine, has to be temporarily removed from the system to undergo permanent magnetic encoding. A continuous bias can also be provided by embedding a suitable arrangement permanent magnets (PMs) [5,6].

Another option is to provide a magnetic bias using an external energy source. The typical configuration of the sensing system is composed of an excitation coil driven by an alternating current (AC), which produces a magnetic flux density distribution whose flux lines take place in the tested material. The system also includes at least one sensing coil (more often an array or a grid of sensing coils is used), magnetically coupled to the excitation one: mechanical stress will produce variations in the magnetic properties that affect the coupling coefficient between the excitation and sense coils [7,8,9]. Often, the magnetic field is provided with an external magnetic circuit in order to drive the magnetic flux into the shaft as much as possible, with the aim to maximize the outputs. However, this method results in increasing losses due to the presence of a magnetic circuit; a limited operating frequency due to skin effects and non-linearities; and, finally, an overall bulky system [10,11]. Torque sensing can be realized as well. When a pure torque is applied to a cylindrical shaft, the stress tensor is composed only of the shear stress, which is maximal on the external surface of the shaft. Such shear stress generates magnetic anisotropy in the material. In particular, there is a variation in the magnetic permeability in directions ±45° with respect to the axis of the shaft, where the major stresses (tensile and compressive) are directed [6,8,12]. Based on these observations, the magnetic anisotropy introduced by shear stress can be used to perform a torque measurement.

**Figure 1:**
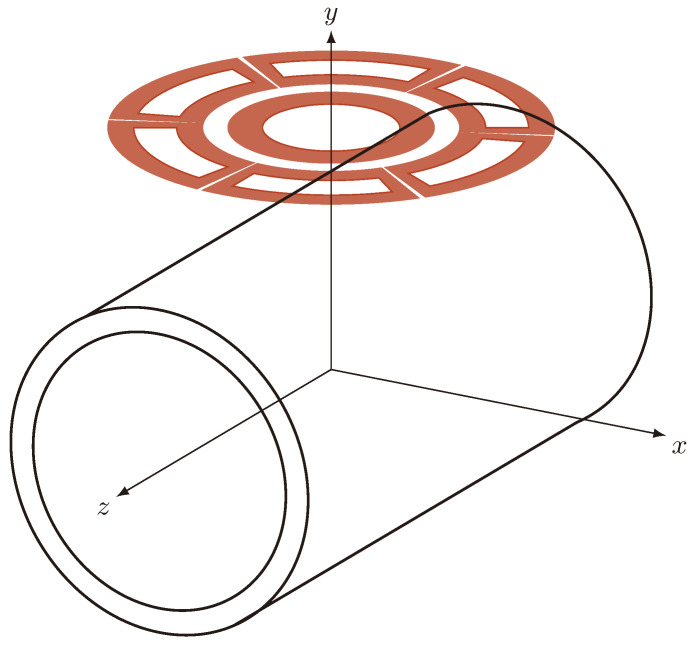
Schematic view of the torque measurement system.

In this manuscript, we propose a new torquemeter able to exploit this anisotropy. It consists of an excitation AC-driven coil coupled with three pairs of detecting coils, whose centers are on the vertices of a hexagon centered on the excitation coil, printed on a tiny circuit board (see Figure 1). This sensor is derived from the one described in [13,14]; its main advantage is the avoiding the interruption of the shaft. The sensing coils, which are opposite with respect to the excitation one, are series connected. The comparison of the voltages detected by the three couples of coils is a measure of the magnetic anisotropy of the material and, consequently, can be used to evaluate the applied torque after a proper calibration. The main advantages of the proposed torquemeter, with respect to the traditional ones discussed above, are then the possibility to have the shaft free from any kind of attached sensors or electronic unit, and consequently the possibility to use the method also in a dynamic rotating shaft. Moreover, the sensor does not need an external iron magnetic circuit to provide the excitation to the shaft; this drastically reduces the complexity and the losses. On the other hand, the main disadvantage of the proposed torquemeter is the need for accurate characterizations of the shaft material from a magnetic and mechanical point of view. This drawback can be overcome with a proper calibration procedure [15,16].

The manuscript is organized as follows: Section 2 briefly reviews the linear magnetoelastic model, while Section 3 describes in detail the sensing system and performs a sensitivity analysis of the position and orientation of the probe with respect to the device under test (an iron shaft), assuming zero torque condition (i.e., isotropic magnetic material); Section 4 reports the main results of a FEM analysis of the system under the action of external torque; and Section 5 describes the measurement setup and reports the results of the experimental tests.

## 2. The Coupled Magnetoelastic Model

Magnetostriction is the change in dimensions of a material due to a variation in its magnetization. This property, which has a quantum–mechanical origin, is exhibited by all magnetic materials to some extent and it is a manifestation of magnetoelastic coupling. The magnetoelastic coupling takes place at the atomic level due to a spin–orbit coupling configuration. From a phenomenological point of view, the material can be assumed to consist of a number of tiny ellipsoidal magnets randomly orientated in a magnetically unexcited system; these magnets rotate and tend to align under the effect of the torque produced by an externally applied magnetic field. The rotation of these elemental magnets produces a dimensional change, leading to free strain in the material [7,17,18,19,20,21].

The inverse magnetostriction effect (or Villari effect) accounts for the change in magnetic properties (e.g., the magnetic permeability) due to mechanical stress induced in the material. The Villari effect has been already exploited in force sensing because of its attractive feature of allowing contactless measurements. The mechanical stress measurement is then reduced to the measurement of the magnetic characteristic of the stressed material [22,23,24,25,26,27,28,29].

Magnetostriction has a non-linear dependence on the magnetic field and mechanical stress induced by the torque applied to the material [5,12,18,19,30]. In particular, magnetic permeability decreases as compressive stress increases, while the B-H curve of the material holds its non-linear behavior. However, if the response of the material consists of small deviations around an operating point far enough from saturation, the effects can be modeled using linear coupled constitutive equations [31]. The relationship between the stress tensor *S*, strain tensor ε, magnetic field vector $\vec{H}$, and magnetic flux density vector $\vec{B}$ can be expressed as follows:

(1) $$\left\{\begin{array}{c} \varepsilon =s_{H}\;S+d_{HT}^{T}\;\vec{H}\\ \vec{B}=d_{HT}\;S+\mu_{0}\mu_{r_{T}}\;\vec{H} \end{array}\right.$$

where $\mu_{0}$ is the vacuum magnetic permeability, $s_{H}$ is the compliance matrices measured at constant magnetic field, and $\mu_{r_{T}}$ is the relative magnetic permeabilities measured at constant stress, while the matrix $d_{HT}$ is the piezomagnetic coupling matrix.

The stress tensor *S* is described by the following matrix:

(2) $$S=\left[\begin{array}{ccc} \sigma_{xx} & \tau_{xy} & \tau_{xz}\\ \tau_{yx} & \sigma_{yy} & \tau_{yz}\\ \tau_{zx} & \tau_{zy} & \sigma_{zz} \end{array}\right]$$

where $\tau_{xy}=\tau_{yx}$, $\tau_{xz}=\tau_{zx}$, and $\tau_{yz}=\tau_{zy}$. The terms of the $d_{HT}$ matrix can be suitably obtained with experimental tests.

For sensing applications and when the elongation strain contribution due to the applied field is negligible, such as for the proposed contactless torquemeter, only the linear equation regarding magnetic flux density vector could be used. In particular, for a crystalline material with tetragonal symmetry, such as the material used in most mechanical engineering applications, the stress–magnetization constitutive relations can be written in the following form:

(3) $$\left[\begin{array}{c} B_{1}\\ B_{2}\\ B_{3} \end{array}\right]=\left[\begin{array}{cccccc} 0 & 0 & 0 & 0 & d_{15} & 0\\ 0 & 0 & 0 & d_{15} & 0 & 0\\ -\frac{d_{33}}{2} & -\frac{d_{33}}{2} & d_{33} & 0 & 0 & 0 \end{array}\right]\;\left[\begin{array}{c} S_{11}\\ S_{22}\\ S_{33}\\ S_{23}\\ S_{13}\\ S_{12} \end{array}\right]+\mu_{0}\;\left[\begin{array}{ccc} \mu_{r_{11}} & 0 & 0\\ 0 & \mu_{r_{22}} & 0\\ 0 & 0 & \mu_{r_{33}} \end{array}\right]\;\left[\begin{array}{c} H_{1}\\ H_{2}\\ H_{3} \end{array}\right]$$

where the subscripts correspond to the spatial reference triad of the *x*, *y*, and *z* axes.

The Voigt notation for an anisotropic material allows the following correspondence:

(4) $$S=\left[\begin{array}{ccc} \sigma_{xx} & \tau_{xy} & \tau_{xz}\\ \tau_{yx} & \sigma_{yy} & \tau_{yz}\\ \tau_{zx} & \tau_{zy} & \sigma_{zz} \end{array}\right]\;\Rightarrow \;S=\left[\begin{array}{c} \sigma_{xx}\\ \sigma_{yy}\\ \sigma_{zz}\\ \tau_{yz}\\ \tau_{xz}\\ \tau_{xy} \end{array}\right]$$

where $\tau_{xy}=\tau_{yx}$, $\tau_{xz}=\tau_{zx}$, and $\tau_{yz}=\tau_{zy}$.

The governing equations are completed by the force balance conditions under static equilibrium:

(5) $$\nabla \cdot S+{\vec{F}}_{V}=0$$

where the ${\vec{F}}_{V}$ are the applied external forces.

The shear stress $\tau_{xz}$, or $\tau_{yz}$ (depending on the reference axis system), is the only component of the stress tensor when a pure torque is applied to a shaft, and it is maximal on the external surface. In particular, its value is

(6) $$\tau_{max}=\frac{2\;M_{T}\;R_{ext}}{\pi \left(R_{ext}^{4}-R_{int}^{4}\right)}=\frac{16\;M_{T}\;D_{ext}}{\pi \left(D_{ext}^{4}-D_{int}^{4}\right)}$$

where $M_{T}$ is the applied torsional momentum, $R_{ext}$ and $D_{ext}$ are the external radius and diameter, and $R_{int}$ and $D_{int}$ are the internal radius and diameter, respectively. The shaft thus undergoes a torsional deformation: the area at the two extremities rotates at an angle that depends on the magnitude of the applied torsional input.

The terms of the piezomagnetic coupling matrix consider the contribution to the magnetic flux variation caused by the shear stress arising from the applied torque. Additionally, any additional mechanical loads placed on the shaft, such as normal stress or bending momentum (i.e., spurious components), result in additional terms in the stress tensor S and are properly considered in the piezomagnetic matrix $d_{HT}$ and in the second equation of system (1). The degree of anisotropy is a measure of the applied shear stress.

## 3. Preliminary Analysis under No-Load Condition

### 3.1. Sensing System Description

**Figure 2:**
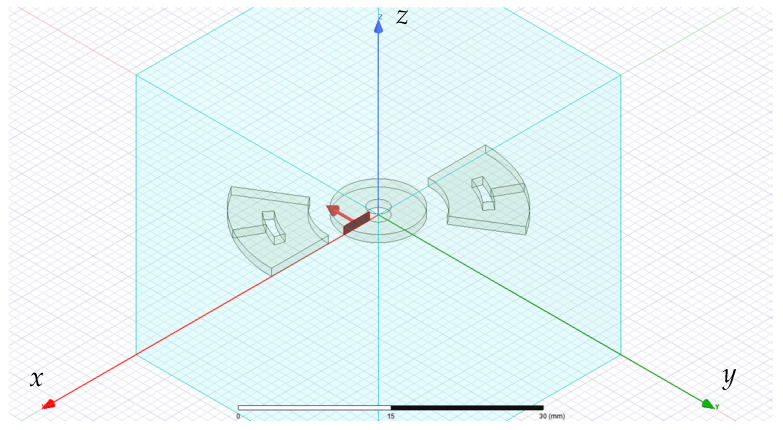
Ampere-turn equivalent model of a sensor with an excitation coil and a single couple of sense coils.

Figure 1 shows the schematic arrangement of the excitation and sense coils. The excitation coil consists of a circular winding with $N_{ex}$ turns fed by a sinusoidal current at 100 kHz and a total magnetomotive force of 22 ampere-turns. The choice of the magnetomotive force is related to the maximum current density in the excitation coil’s conductors while limiting its dimensions to reduce the leakage fluxes. The working frequency, instead, is a compromise between the maximization of the voltage induced in the sensing coils and the limitation of the skin effect inside the shaft. Six identical sensing coils, with $N_{sense}$ turns each, are positioned around the excitation coil. A couple of sense coils that are opposite with respect to the excitation one are series connected with opposite magnetic flux to form three sense windings. Considering the position of the sense coils and their connections, the voltage induced in a sense winding by the excitation coil’s current is the sum of the voltages induced in the two sensing coils composing the winding. Figure 2 shows two sensing coils series connected and placed around the excitation one.

Before starting the investigation of device performance, we carried out a sensitivity analysis to evaluate the influence of the presence of a metallic enclosure and of the sensor’s relative position with respect to a ferromagnetic shaft with no applied torque. To investigate such influences, we derived the self and mutual inductances of the coils and the voltages induced in the sense coils in the different configurations. Preliminary experimental measurements were performed to validate the sensitivity analysis.

Several numerical analyses were performed using the Finite Element package Ansys-Maxwell [32] as well the research code EN4EM (Electric Network 4 Electro-Magnetics) based on Integral Formulation of Maxwell Equations, developed at the DESTEC and previously used to analyze different kind of actuators [33,34]. To reduce the computation time, an ampere-turn-equivalent model was analyzed. The model was built substituting each coil with a single turn with the same cross section as the original coil. The true values of self and mutual inductances were then scaled considering the number of turns.

We started considering the excitation coil and a couple of sensing coils only, oppositely positioned to form a sense winding (see Figure 2), without the ferromagnetic shaft. Simulations on this model provided the following results: $L_{ex,FEM}=37.385\;\mu H$ and $L_{ex,EN4EM}=35.55\;\mu H$; $M_{ex,det,FEM}=2.35\;\mu H$ and $M_{ex,det,EN4EM}=2.18\;\mu H$. Here, $L_{ex}$ is the self-inductance of the excitation coil, and $M_{ex,det}$ is the mutual inductance between the excitation coil and the sense winding. The second part of the subscript identifies the used numerical formulation (FEM or EN4EM).

A measurement campaign was performed in correspondence with the rated magnetomotive force of 22 AT at f = 100 kHz. We obtained $L_{ex}^{meas}≃35.9\;\mu H$ as the self-inductance coefficient of the excitation coil and $M_{ex,det}^{meas}≃2.27\;\mu H$ as the mutual-inductance coefficient between the sensing coil and each couple of sensing coils. We also measured the resistance of the excitation, obtaining $R_{ex}^{meas}≃7.4\;\Omega$.

The system of excitation and sense coils was arranged in an aluminum enclosure used for both the mechanical protection of the coils and the shielding from unwanted stray electromagnetic fields [35]. The enclosure consisted of an aluminum cylinder with an external radius of 20 mm, a thickness of 3 mm and a height of 60 mm; the sensing system was placed on the bottom of the cylinder.

FEM analyses and EN4EM were carried out in this configuration, too. The computed values of the self-inductance coefficient of the excitation coil were $L_{ex,FEM}≃36.7\;\mu H$ and $L_{ex,EN4EM}≃35.4\;\mu H$, while the mutual induction coefficient between the excitation coil and a couple of sense coils results were $M_{ex,det,FEM}=2.78\;\mu H$ and $M_{ex,det,EN4EM}=2.73\;\mu H$. The corresponding measured quantities were $L_{ex}^{meas}=35.9\;\mu H$ and $M_{ex,det}^{meas}≃2.76\;\mu H$. We observed that the presence of the aluminum enclosure had a marginal effect on $L_{ex}$, while producing an increase of about 20% on $M_{ex,det}$. This can be explained assuming that the metallic enclosure acts as a flux concentrator, reducing the flux density outside. This effect was more evident in $M_{ex,det}$ than in $L_{ex}$ since the sense coils were in proximity of the enclosure, while the excitation coil was far enough. This is a positive effect since it improved the sensibility of the device.

**Figure 3:**
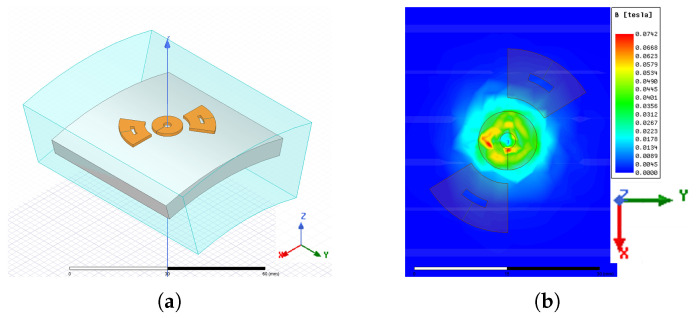
Meshed domain for the FE analysis: shaft portion and air box (**a**), and flux density distribution on the shaft surface (**b**).

Subsequently, at 1 mm distance from the ampere-turn sensor model, a shaft was inserted. Its dimensions were $r_{int}=93\;\mathrm{mm}$, $r_{ext}=99\;\mathrm{mm}$, $\ell_{shaft}=400\;\mathrm{mm}$. The material of the shaft was structural steel, with isotropic magnetic properties and the following parameters: $\mu_{r}=500$, $\sigma =4.03\times 10^{6}\;S/m$. We considered only the excitation and sensing coils discarding the enclosure. Since the dimensions of the shaft were very large with respect to the coils, we checked the possibility of simulating only the region of the shaft near the sensor. We progressively reduced the meshed portion of the shaft, checking the consistency of the results (in terms of magnetic flux density distribution) by comparing the distribution obtained from a reduced geometry with the one produced in the complete model. After several simulations, we derived the results visible in Figure 3.

**Figure 4:**
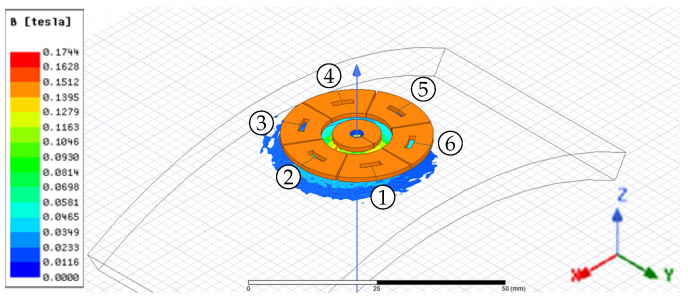
Complete sensor over a ferromagnetic shaft in air. The excitation and sensing coils layout over the under-testing shaft is visible. The pickup coils 1 and 4, 2 and 5, and 3 and 6 are electrically connected in series.

The same simulation parameters were used to analyze the complete sensing system, composed of one excitation coil and six sensing coils around it. Its finite element model and magnetic flux density distribution are visible in Figure 4.

### 3.2. Sensitivity Analysis

Since the shaft is part of the torquemeter, its response is influenced by displacements and possible misalignments in the shaft with respect to its axis. For this reason, we performed a sensitivity analysis with respect to parameters describing the relative position between the sensor and the shaft. In particular, we considered the gap along the z-direction between the sensor and the shaft, the pitch angle (i.e., the rotation angle of the sensor with respect to the x-axis), and the displacement of the sensor along the x-direction and the yaw angle (i.e., rotation angle with respect to the z-axis) of the coil system.

**Figure 5:**
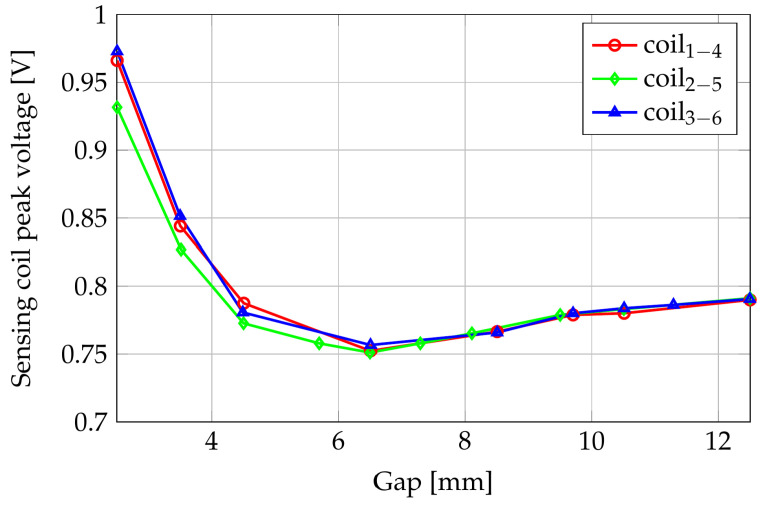
Amplitudes of the induced voltages as functions of the gap.

Figure 5 shows the variation in the induced voltage amplitude with the gap along the z-direction between the sensor and the shaft sweeping from 2.5 mm to 12.5 mm with a step of 1 mm (assuming that the shaft is made by isotropic material with the parameters above reported).

For gaps smaller than 6 mm, the voltage induced in the couple of coils 2–5 was lower than $V_{14}$ and $V_{36}$. This behavior is due to the position of such coils; the magnetic flux lines crossing them follow the maximum air gap path, and the induced voltage results in the lower one. When the distance from the shaft increases, the differences between the paths in air lower, and the induced voltages become coincident.

**Figure 6:**
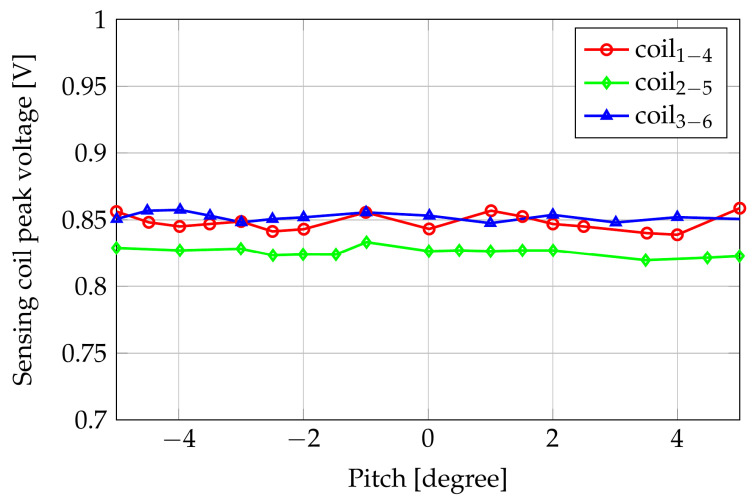
Amplitudes of the induced voltages with respect to the pitch angle.

Moreover, we considered the sensitivity with respect to the pitch angle. In this case, the gap (as in the following simulations for the sensitivity analysis) was 3.5 mm. Figure 6 shows the results in terms of the amplitude of voltage induced in the three couple of coils in correspondence with a pitch angle sweeping from $-5^{\circ }$ to 5° with 1° steps.

**Figure 7:**
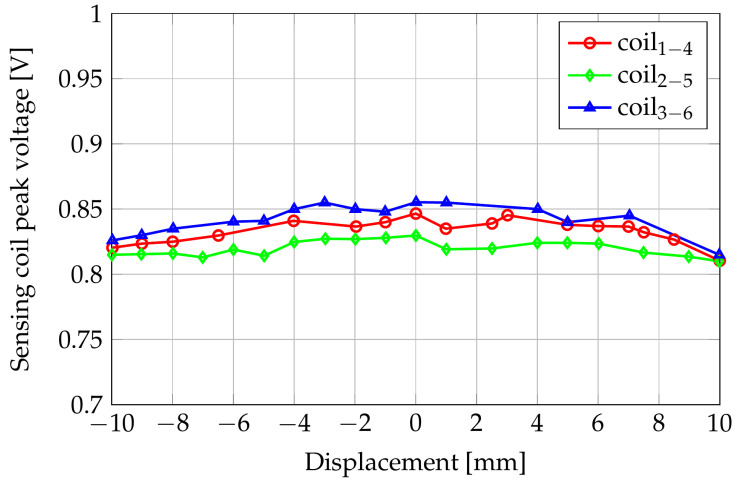
Amplitudes of the induced voltages with respect to the displacement along the x-axis.

We also considered the variation of the induced voltages when the sensor moves transversely with respect to the shaft along the x-axis on the XY plane. Figure 7 shows the results for a sweeping from −10 to 10 mm with 1 mm steps. The gap corresponding to the nominal position (zero lateral displacement) is 3.5 mm.

**Figure 8:**
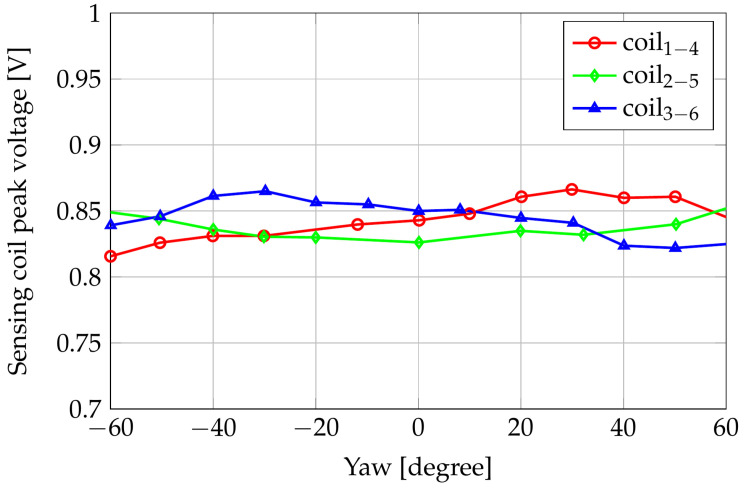
Amplitudes of the induced voltages with respect to the yaw angle.

Finally, we considered the rotation angle of the sensor with respect to the z-axis. Figure 8 shows the corresponding variations of the induced voltages for a yaw angle sweeping from $-60^{\circ }$ to 60° with 10° steps. Values of the sensitivity for angles outside the above interval can be obtained by the sensitivity of other sensing coils. Considering the coils 1–4 as an example, we observed that a rotation of 60° moved these coils on the initial position of coils 3–6.

From this sensitivity analysis, it is possible to derive that the most significant variation of the induced voltages is the one relative to the gap. The influence of the pitch is marginal, while, as expected, the maxima of the induced voltages with respect to the displacement are in correspondence with null displacement. Furthermore, it is interesting to observe how a yaw angle of 60° produces a cyclic permutation of the induced voltages. This behavior was investigated deeply, and further measurements over 360° yaw rotation are shown in the next section.

## 4. Numerical Modeling of the Torquemeter

**Figure 9:**
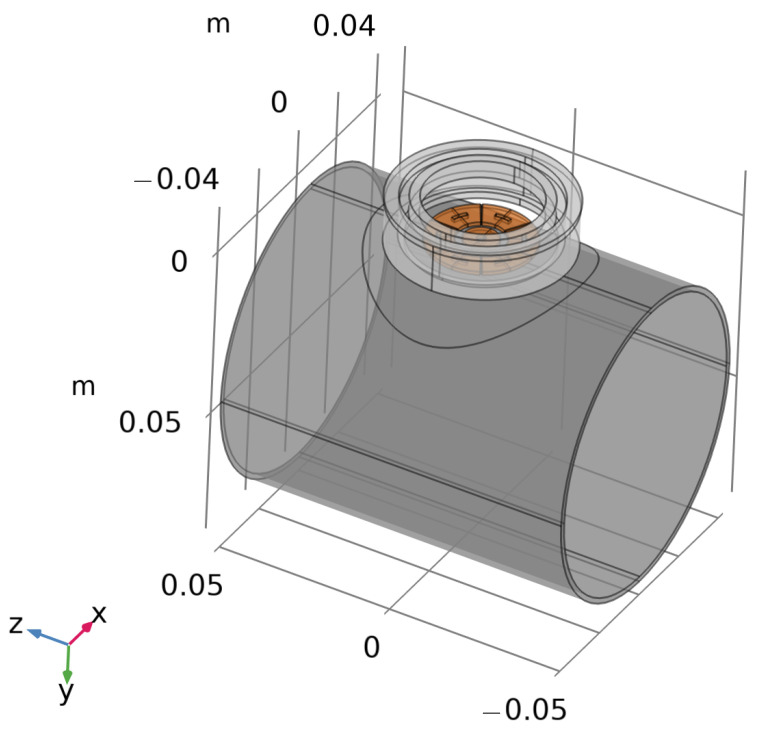
FE model of the analyzed geometry.

The coupled problem, described in Section 2, was modeled by the COMSOL Multiphysics Suite [36] using three modules: Solid Mechanics, Magnetic Field, and Electrical Circuit. The meshed domain was constituted by the shaft, the excitation and sensing coils, the metallic enclosure, and the surrounding air portion (see Figure 9).

For the mechanical (elastic) model, to simplify the analysis, a fixed constraint was applied at one end of the shaft (i.e., the displacement of the shaft is null), while, at the other extremity, a pure static torque was set. Other effects, such as the influence of bending or normal stress, were neglected in this study. The shaft was treated as a linear magnetostrictive material, in which only the Villari effect was considered (refer to Equation (1)) in the Solid Mechanics and Magnetic Field solvers.

The model described by Equation (3) requires five unknown parameters (namely, $\mu_{r_{11}}$, $\mu_{r_{22}}$, $\mu_{r_{33}}$, $d_{15}$ and $d_{33}$), which we estimated by performing several preliminary numerical simulations and experimental analyses. For the shaft material, the mechanical steel S235 was considered [37], modeling it as a linear material since the values of the magnetic flux density were low. Moreover, we took into account the manufacturing process of the shaft itself: since it is a hollow cylinder, we considered that it was manufactured by tube rolling and welding. This manufacturing process results in different residual stresses along the axial and circumferential direction [38], which result in anisotropic magnetic characteristics of the shaft material along the three directions. This behavior was modeled in the FE analysis by imposing for the material of the shaft the three relative magnetic permeability, $\mu_{r_{11}}=300$, $\mu_{r_{22}}=300$, and $\mu_{r_{33}}=200$ along the radial, circumferential and axial directions of a cylindrical reference system centered on the shaft axis.

Concerning the two magnetomechanical parameters (i.e., $d_{15}$ and $d_{33}$), they were estimated by several “multi-objective” minimization processes aimed to reduce the differences between the experimental data and numerical simulations, both obtained by applying different known torque values. The derived values were $d_{15}=25\times 10^{-9}\;m/A$ and $d_{33}=8\times 10^{-9}\;m/A$, compatible with those found in the literature and related to iron alloys for mechanical applications [39,40,41].

We started the numerical simulations considering the linear system above described, loaded by a pure torque ranging from 0 Nm to 1200 Nm. The geometry of the excitation and sensing coils was unchanged (see Figure 4), while the shaft was a hollow cylinder with an external diameter of 85 mm and an internal one of 82 mm. The total axial length was 400 mm.

**Figure 10:**
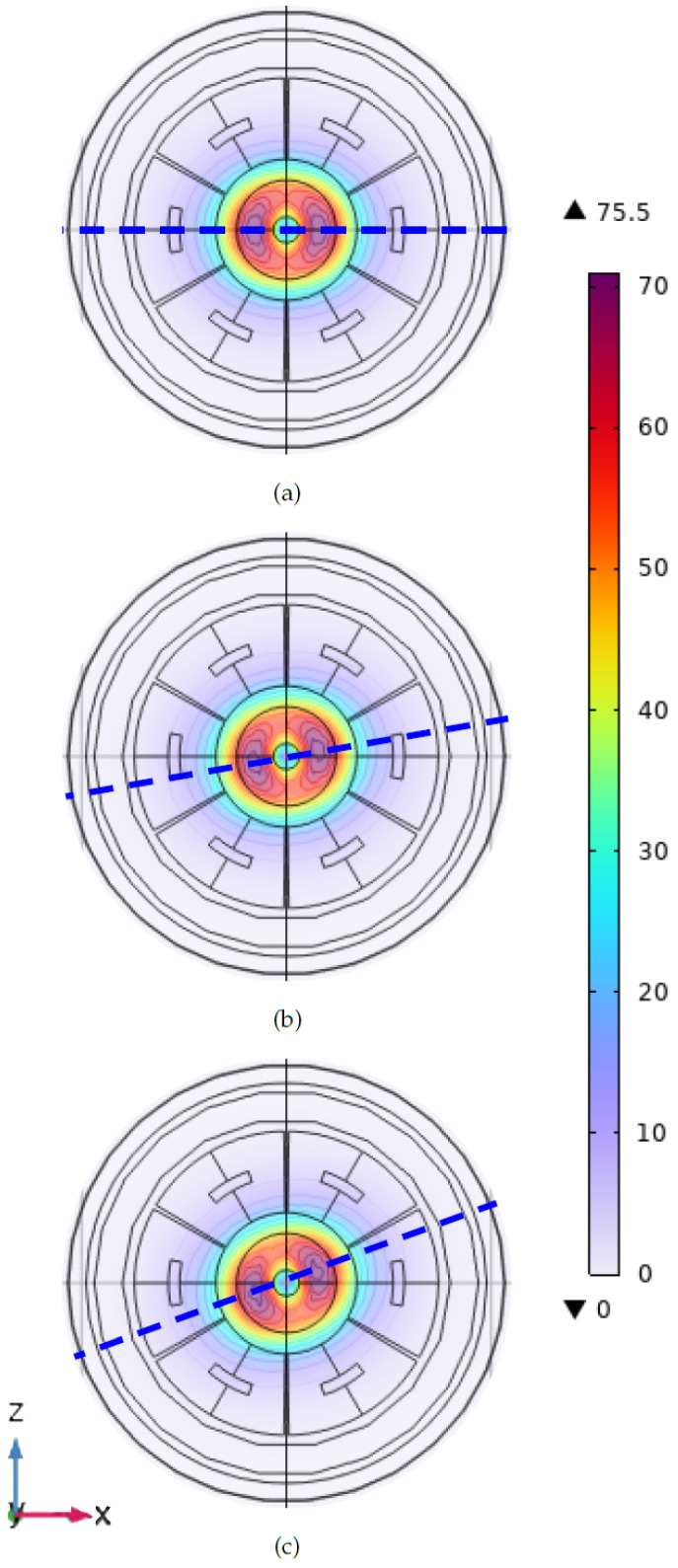
Amplitude of the magnetic flux density in the 3D FE model at 0 Nm (**a**), 600 Nm (**b**), and 1200 Nm (**c**). Values are expressed in milliTesla.

Figure 10 reports the amplitude of the magnetic flux density distribution on the shaft’s surface for a pure applied torque of 0 Nm, 600 Nm, and 1200 Nm. It is possible to observe the magnetic flux density distribution near the excitation coil. Such an induction affects the magnetic flux variation detected by the three couples of sensing coils. The magnetic flux density amplitude increases with the applied torque, and the regions of maximum B amplitude align along the most solicited fibers, in agreement with the theoretical modeling. The shear stress produces a magnetic response in terms of anisotropy that is properly detected using the variations of the voltages induced on the sense coils with respect to the unloaded condition.

**Figure 11:**
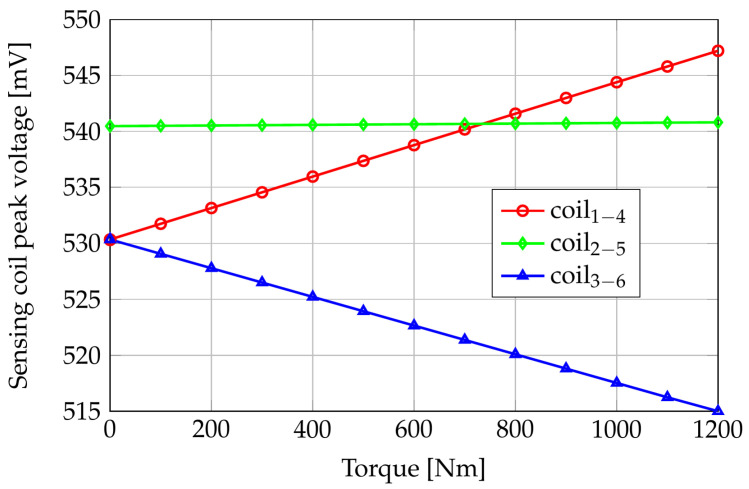
FE computed sensing coils peak voltages versus applied pure torque.

**Figure 12:**
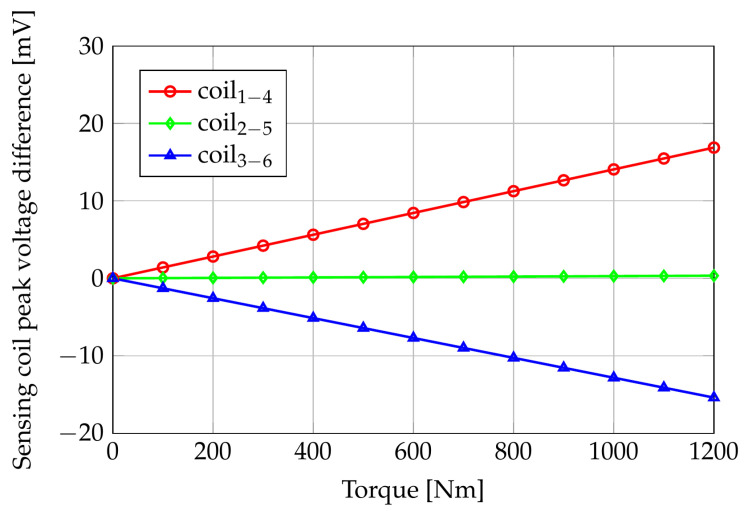
FE computed sensing coils peak voltage difference (with respect to torque = 0 Nm) versus applied pure torque.

Because of the assumed system linearity, the voltages induced in the sense coils are sinusoidal waveforms whose peak values depend on the applied torque. Figure 11 shows the FE computed sensing coil peak voltages versus the applied torque, while Figure 12 reports the differences of the same quantities with respect to their null torque initial values.

Looking at Figure 11, the maximum peak voltage at null torque is on coils 2–5, which are perpendicular to the axis of the shaft; the path in the air of the flux lines is maximal for these coils, but the magnetic anisotropy of the shaft material results in a reduced reluctance of the azimuth paths with respect to the axial ones. As a consequence, the flux linkage of coils 2–5 is greater than the ones in coils 1–4 and 3–6. This result highlights the importance of a correct magnetomechanical characterization of the shaft material: considering a shaft without residual mechanical stresses (i.e., magnetically isotropic), the sensing coil peak voltage of coils 2–5 would be about 520 mV, slightly smaller than $V_{14}$ and $V_{36}$. In zero-torque conditions, the voltages induced in coils 1–4 and 3–6 are the same because of their symmetric position.

Coils 1–4 peak voltage increases by about 17 mV in the applied torque range because of their partial alignment with the tensile stress direction. Coils 3–6, instead, are partially aligned with the compressive stress direction, and the peak value of $V_{36}$ shows a decrement of about 15 mV when the applied torque increases from 0 Nm to 1200 Nm. Coils 2–5 are aligned with the bisector of the tensile and compressive directions, and their induced voltages have an almost constant behavior with respect to the applied torque.

## 5. Experimental Validation

### 5.1. Experimental Setup

This section describes some of the tests carried out on a sample shaft using the equipment available in the DESTEC laboratories.

**Figure 13:**
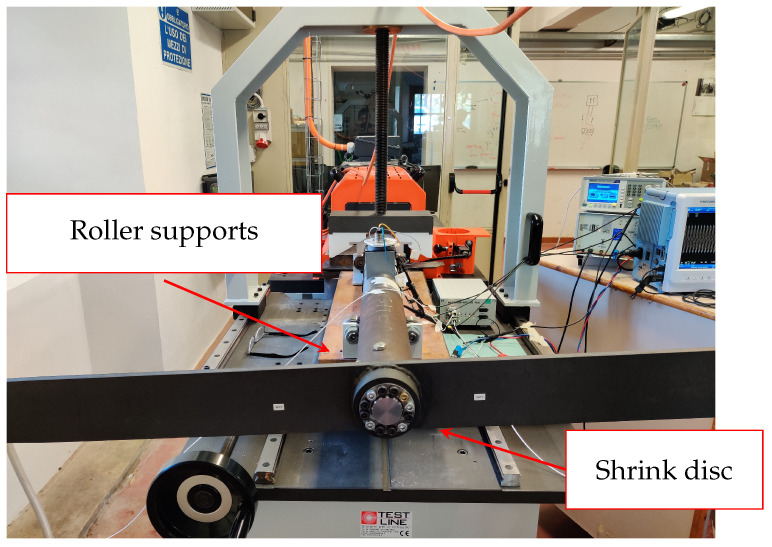
Sample shaft used to test the proposed system coupled to a 2 m long bar and a test rig.

**Figure 14:**
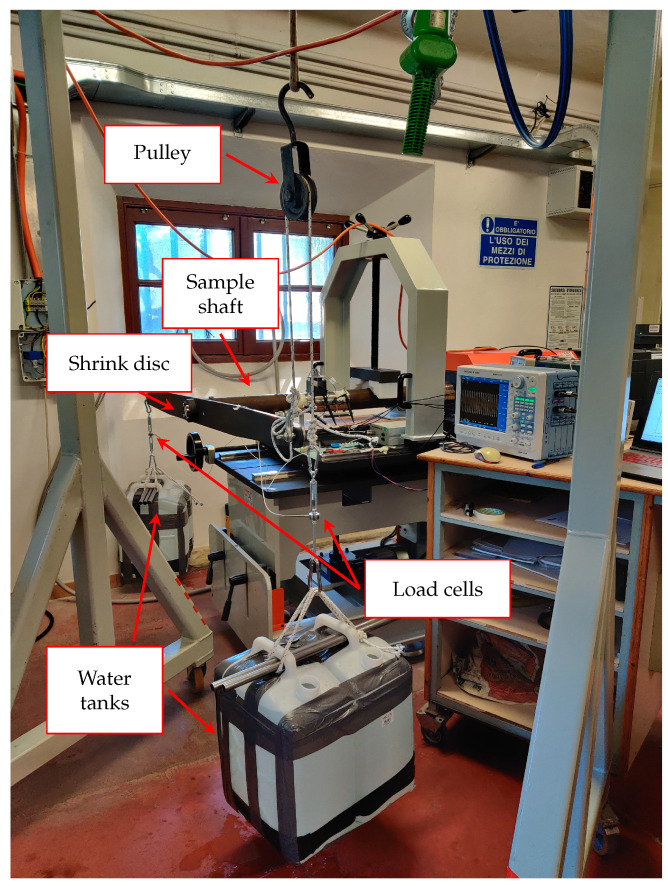
Experimental setup used to produce the static mechanical excitation.

To investigate the proposed system, we used a sample shaft with an external diameter of 85 mm and an interior one of 82 mm, made of structural steel S235 (ex. Fe 360). The shaft was coupled to a 2 m long bar at one extremity through a shrink disc and was fixed at the other using a mechanical plug inserted in the test rig frame. The Unipi team designed and realized both mechanical joints customized for the chosen shaft (see Figure 13 and Figure 14).

The shaft was loaded with water tanks placed at both ends of the bar connected with the shrink disc. The torque applied to the shaft could be controlled by changing the weight of the tanks. Figure 14 depicts the described setup which also comprised a pulley used to produce a pure torque.

**Figure 15:**
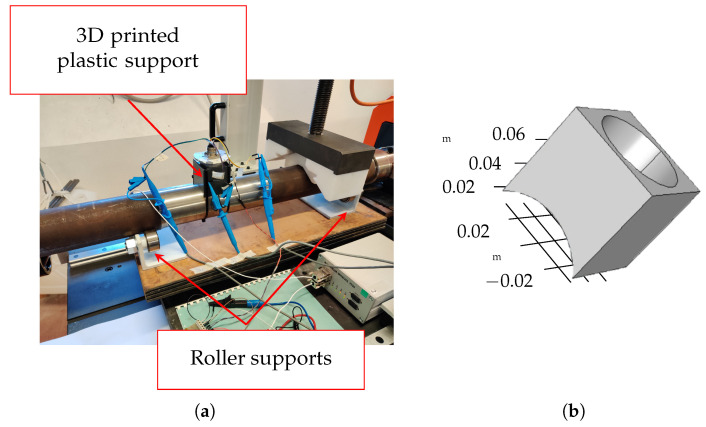
Sample shaft under test with mounted probehead (**a**) and 3D printed probehead support (**b**).

The sensing system (i.e., excitation and sensing coils) was fixed in the middle of the shaft with a 3D-printed support (see Figure 15). The distance between the probehead and the shaft could be varied with the insertion of spacers in the bottom part of the support. In the measurement campaign, the gap between the sensing system and the shaft was 3.5 mm.

It is possible to have two different setup configurations depending on the weights applied to the bar extremities:

1. One or more equal water tanks are applied to both ends;
2. The water tanks are applied only at one end of the bar.

In the first case, a pure torque is applied to the shaft: the weight force of the water tanks at one end of the bar is exerted in the downward direction, while, thanks to a pulley, the weight force of the other tanks, at the opposite end, is upward directed. In this way, the applied load is a pure torque, and the possibility of bending is strongly reduced since the only bending load is the bar weight. In the second case, instead, the applied torque is not pure because, in addition to the torsional load, a bending moment is also present. Such a moment results from the unbalanced weight forces of the water tank added to the bar weight force. The customized setup can apply up to 1200 Nm in pure static torque conditions and a maximum of 600 Nm in non-pure torque conditions.

The tests consisted of the measurement of the detected pick-up coil voltages induced by the excitation current at different applied static loads. Ten consecutive peaks of the sensing coil voltages were measured once the system had provided a stable measurement and any possible transient effect was extinguished. The average value and standard deviation were computed to have a good level of confidence. The measurement setup was constituted of the following instrumentation:

- Oscilloscope Yokogawa DL850E;
- Waveform signal generator Tektronix AFG 1022;
- Power amplifier Toellner TOE 7610;
- Two load cells Burster 8431-6002 (2 kN each cell) connected to a PC through a data acquisition board;
- customized PCB with 1 Ω precision resistor to measure excitation current.

### 5.2. Pure Torque Tests

**Figure 16:**
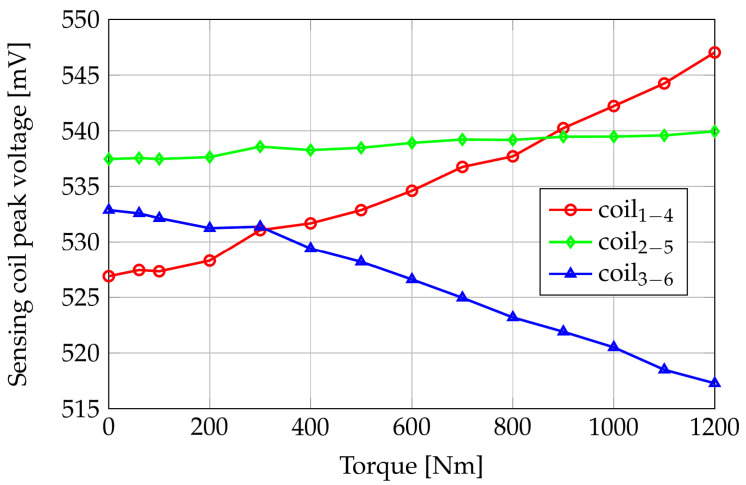
Peak voltages on the sensing coils as a function of applied pure torque. Experimental results.

Figure 16 reports the sensing coils’ peak voltages versus applied pure torque in the range [0; 1200] Nm obtained by water-filling two tanks of 30 L each at both ends of the bar. The effective weight, and then the applied torque, is measured by two load cells. It is worth noting that, in the unloaded condition, the induced voltage in the three sense coils is not the same because of different air gaps between the coils and the shaft. Moreover, the unavoidable imperfections and the magnetic anisotropy of the material given by the manufacturing process of the shaft and the tolerances on the holder realization cause differences in the magnetic paths seen by the three couples of coils, resulting in different induced voltages, also in unloaded conditions. As expected, coils 2–5 have the highest no-load induced voltage. Coils 1–4 and 3–6, which are expected to have the same no-load voltages, show a difference that may depend on the variability in the manufacturing process of the sensor, on the tolerance on the 3D-printed plastic support, and on the presence of residual stresses in the shaft material which produce magnetic anisotropies.

**Figure 17:**
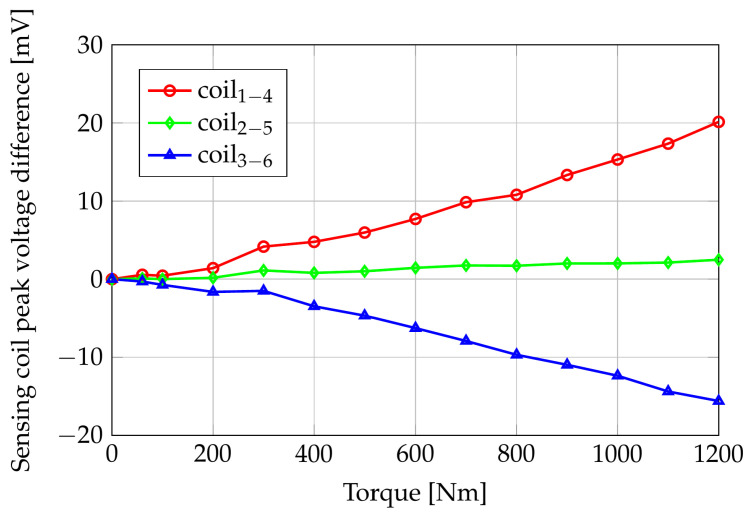
Sensing coils peak voltage difference (with respect to null torque) versus applied pure torque. Experimental results.

Considering the decomposition of the torsional stress in tensile and compressive stresses along orthogonal directions, it is possible to note that the coils oriented along the stretched fibers of the shaft (tensile condition, i.e., coils 1–4) show an increasing peak voltage for increasing torque values. Conversely, the peak voltage of coils 3–6, oriented along the compressed fiber, decreases with respect to the applied torque. This behavior agrees with the inverse magnetostrictive effect and the theoretical part discussed in the previous sections. Coils 2–5 are oriented perpendicularly to the shaft axis, and their peak voltage, as expected, is marginally affected by the torque with respect to the other two couples of coils. The effectiveness of the contactless sensor is better appreciated by looking at Figure 17, which reports the difference between the induced voltages under load and the voltages in the unloaded case (i.e., zero applied torque). The sensing characteristic is roughly linear with the applied torque in the considered range, with an increase over the applied torque interval of about 20 mV for the sensing coils 1–4 (gain $g_{1-4,meas}=\Delta V_{1-4,meas}⁄\Delta T=0.0167\;\mathrm{mV}⁄\mathrm{Nm}$), corresponding to stretched fibers/tensile condition, and a decrease over the applied torque interval of about 15 mV for the sensing coils 3–6 (gain $g_{3-6,meas}=\Delta V_{3-6,meas}⁄\Delta T=-0.0125\;\mathrm{mV}⁄\mathrm{Nm}$) for the compressed fibers/compressive condition. The gain of the couple 2–5 is about one order of magnitude smaller than those in the other directions since, as already observed, these coils are directed along the bisector of the tensile and compressive stresses.

If we compare the measured gains of coils 1–4 and coils 3–6 with those predicted by the numerical model and reported in Figure 12 ($g_{1-4,sim}=0.0142\;\mathrm{mV}⁄\mathrm{Nm}$, $g_{3-6,sim}=-0.0125\;\mathrm{mV}⁄\mathrm{Nm}$), we observe that the ratios between the simulated and measured gains for the two directions are in good agreement: $g_{1-4,sim}⁄$$g_{1-4,meas}=0.85$, $g_{3-6,sim}⁄$$g_{3-6,meas}≃1$. The difference between the computed and the measured gains, in particular, for coils 1–4, can be attributed to errors in the characterization of the shaft material, geometry of the sensor, and position of the sensor with respect to the shaft. However, once the characteristic of the sensor has been experimentally obtained, the difference between measurements can be corrected in the calibration steps.

It is worth observing that coils 2–5 show an increase in the detected peak voltage. This behavior could be caused by a non-perfect alignment of the PCB coils into the aluminum housing and/or residual stress in the shaft due to the rolling manufacturing process.

### 5.3. Non-Pure Torque Tests

**Figure 18:**
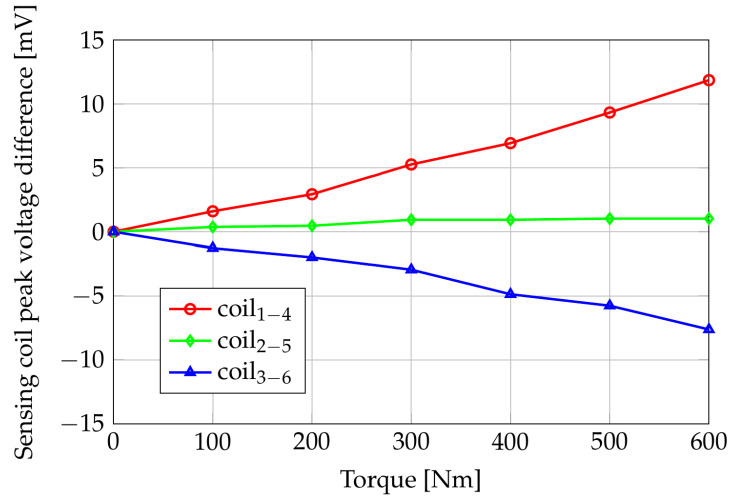
Sensing coils peak voltage difference (with respect to null load) versus applied torque in case of non-pure torque test. Experimental results.

In these tests, water tanks are applied only at one end of the 2 m long bar, then both torque and bending are applied to the shaft. Results of the measurements are shown in Figure 18. The values are reported with respect to the unloaded condition (torque = 0 Nm).

The gains over the applied torque interval are $g_{1-4,npt}=0.0198\;\mathrm{mV}⁄\mathrm{Nm}$ and $g_{3-6,npt}=0.0127\;\mathrm{mV}⁄\mathrm{Nm}$. The ratio between them, $g_{1-4,npt}⁄$$g_{3-6,npt}=1.56$, is greater than the ratio $g_{1-4,meas}⁄$$g_{3-6,meas}=1.34$ evaluated when a pure torque is applied. This is a consequence of the bending moment caused by the non-pure torque applied to the shaft. This produces tensile stresses on the upper part of the horizontal shaft in correspondence with the sensor. The fibers oriented according to the coils 1–4 undergo an increase in tensile stress with a consequent rise in the induced voltage for the same applied torque. This produces an increase in the “sensitivity” of coils 1–4. Similar considerations applied to coils 3–6 lead to a decrease in their sensitivity, which, however, does not appear as evident as the increase in the sensitivity of coils 1–4.

Finally, considering the voltage on coils 2–5, we observe a small positive gain due to the shaft bending, which produces tensile stress on the shaft portion under the sensor. As observed above, the torque marginally affects the detected voltage because of the alignment of coils 2–5 with a direction orthogonal to the shaft axis.

### 5.4. Loading and Unloading Measurements

**Figure 19:**
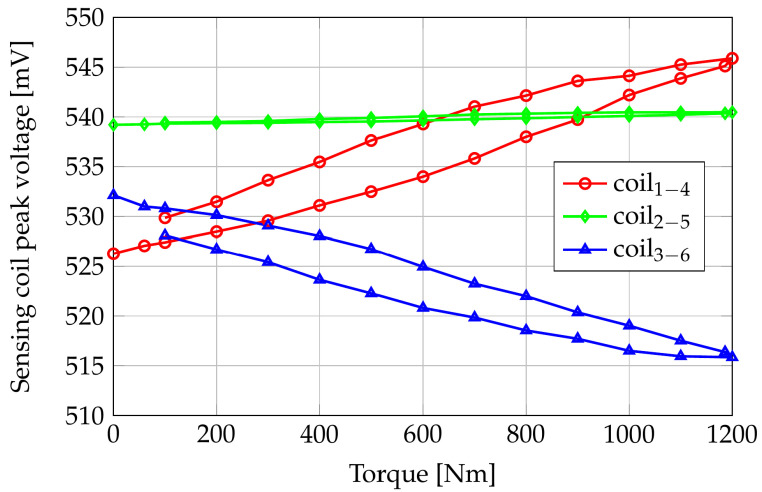
Sensing coils peak voltage versus applied torque: loading and unloading cycle. Experimental results.

In this section, experimental tests with variable applied torque are shown. In particular, we investigate the behavior of the sensor in a torque cycle by loading and unloading the shaft. Firstly, the water tanks are filled, and once the maximum levels are reached, the water is pumped out from the tanks. Figure 19 reports the measurement results that show a hysteretic-like behavior given by the magnetomechanical characteristics of the shaft’s material. In applications with strict requirements (e.g., aerospace applications), the magnetomechanical characteristics of the shaft must be properly investigated, and if the chosen material shows this hysteretic effect, it should be properly considered in the calibration and measurement processes. Conversely, it is necessary to choose a shaft with a lower hysteretic effect.

### 5.5. Experimental Sensitivity Analysis

The measurement campaign was concluded by checking the proper alignment of the sensing coils with the axis of the shaft. As is known, the stress due to a pure applied torque results in tensile and compressive stresses in directions sloped by ±45° with respect to the axis of the shaft. As a consequence, the sensitivity of the measured voltages with respect to the relative orientation of the sensor and the shaft (i.e., the yaw angle) assumes great importance.

**Figure 20:**
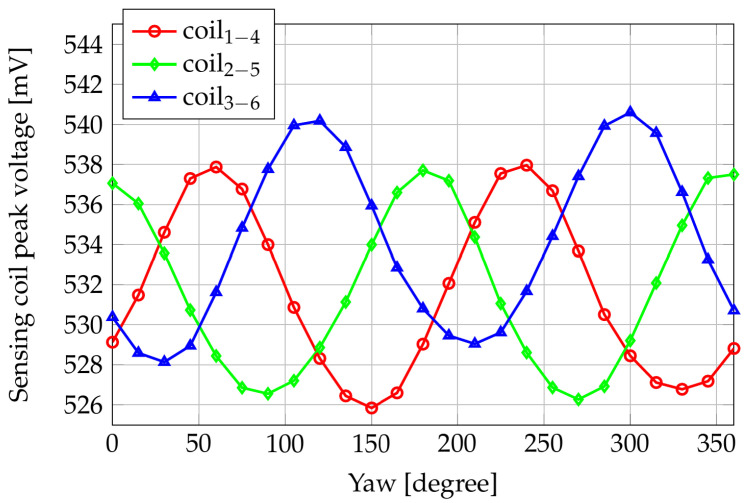
Sensing coils peak voltages versus yaw angles, in unloaded shaft conditions. Experimental results.

The sensor was placed 3.5 mm above the shaft, varying its yaw angle. A 360° protractor was placed on a 3D-printed plastic support, and the probe was clockwise rotated with a step of 15°. Figure 20 shows the sensing coils’ peak voltages versus the yaw angle corresponding to the rated magnetomotive force in the excitation coil and an unloaded shaft (i.e., no torque and/or other mechanical input are applied). The null angle corresponds to coils 2–5 oriented perpendicularly to the shaft axis (i.e., the configuration shown in Figure 4), while 90° corresponds to coils 2–5 aligned to the shaft axis.

It is noticeable that the periodicity of the three couples of sensing coils is due to the symmetry of the arrangement. By comparing the sensed peak voltages of coils 1–4 and 3–6, it is possible to note that such coils behave in the same way with a yaw interval difference of 60°. Indeed, with a geometrical rotation of 60° in the clockwise direction, coils 1–4 are located in the initial position (i.e., yaw angle = 0°) of coils 3–6. Furthermore, when the couple 2–5 is in the perpendicular direction with respect to the shaft axis (i.e., yaw angle 0° or 180°), the magnetic flux lines from the excitation coil to the sense ones take the longest magnetic path (air gap) through the shaft. However, because of the magnetic anisotropy of the shaft material, $V_{25}$ results in the highest value as previously discussed. Oppositely, when the yaw angle is 90° or 270°, the voltage on coils 2–5 is at its minimum. Analogously, the peak of $V_{14}$ occurs with a rotation of 60°, i.e., when the couple 1–4 is located in the initial position of 2–5.

The waveform of the voltage induced in the couple 3–6 is different from the one in 2–5 and 1–4; this confirms some variability in the manufacturing process of the coils.

## 6. Discussion and Conclusions

In this paper, the performance of a contactless torquemeter based on the inverse magnetostrictive effect has been analyzed and tested. The proposed transducer does not need to introduce any discontinuity on the (ferromagnetic) shaft as many conventional torquemeters do. Once the calibration algorithm is properly tuned, the contactless inverse magnetostrictive torquemeter may represent a reliable, economical, and easy-to-install device.

Based on the theoretical, numerical, and experimental investigation performed on the first prototype, it is possible to conclude that the sensor can detect the change in the magnetic properties of a magnetic shaft due to applied mechanical stresses, such as pure torque, by detecting a variation in the induced voltages. The theoretical analysis and the experimental results have confirmed the capability of the sensor and its potential application in transmission shafts. However, the studies highlighted some drawbacks, which may affect the accuracy of the measurements. Since both simulations and experiments have confirmed the sensitivity of the sensor response to possible misalignments, a system that accurately aligns the sensor with the axis of the shaft is needed. In addition, the sensor has also shown (with the material used to manufacture the shaft) a hysteretic behavior under loading and unloading test conditions. This requires a remarkable effort in software development for processing raw measured data with the capability of performing a suitable calibration and correcting different errors (e.g., misalignment error, temperature changes, and hysteresis) to obtain better and repeatable measurements. Moreover, since the characteristics of the shaft manufacture strongly affect the torquemeter response, a preliminary characterization campaign has to be performed to obtain the magnetomechanical properties of the material. In this framework, the improvements of the device’s performance in terms of sensitivity and accuracy are related to the increase in the amplitude of the induced voltages and their correlation with the applied torque. To this aim, it is necessary to investigate the use of the shaft’s materials with increasing magnetostrictive properties or different configurations by changing the sensor dimensions, geometry, displacements, and number of excitation and sensing coils.

## Data Availability

The datasets presented in this article are not readily available because the data are under a patent pending process. Requests to access the datasets should be directed to Avio Aero, A GE Aerospace company.
